# Iron testes: sperm mitochondria as a context for dissecting iron metabolism

**DOI:** 10.1186/1741-7007-8-79

**Published:** 2010-06-21

**Authors:** Karen G Hales

**Affiliations:** 1 Department of Biology, Davidson College, Box 7118, Davidson, NC 28035, USA

## Abstract

A recent paper in *BMC Developmental Biology *reports that a mitochondrial iron importer is required for *Drosophila *male fertility and normal mitochondrial shaping in spermatids. This suggests that mitochondrial morphogenesis during insect spermatogenesis may be a useful new context in which to study iron metabolism.

See research article http://www.biomedcentral.com/1471-213X/10/68

## Commentary

**Figure 1:**
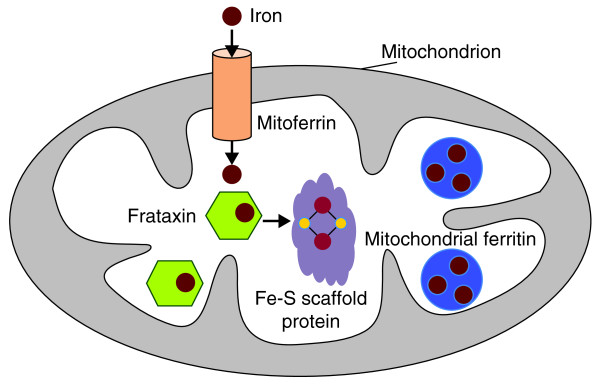
**Many proteins affect iron metabolism within mitochondria**. Members of the mitoferrin family (orange cylinder) mediate iron import into mitochondria. Frataxin family members (green hexagons) bind iron and help deliver it to proteins that assemble Fe-S clusters (purple shape). Other mitochondrial proteins involved in respiration (not shown) incorporate the Fe-S clusters as cofactors. Mitochondrial ferritin (blue circles) and frataxin both contribute to iron sequestration and protection from oxidative damage.

Mechanisms of iron homeostasis have been characterized primarily in vertebrate blood, liver, and intestinal tissue, as well as in budding yeast. In vertebrates, iron transport and trafficking in and out of cells requires such proteins as transferrin, transferrin receptors, ferroportin, hepcidin and ferritin [1]. Once in the cytoplasm, iron relies on other proteins for import into mitochondria and further processing. Iron homeostasis is intimately connected with mitochondrial function. Many electron carriers required for respiration in the inner mitochondrial membrane include iron-sulfur (Fe-S) cluster cofactors, as do two enzymes of the citric acid cycle; furthermore, mitochondria are the site of iron incorporation into heme in erythroid tissue. Protein players in mitochondrial iron transport and metabolism (Figure 1) include mitoferrins (iron transport into mitochondria), frataxins (delivery or iron to Fe-S cluster scaffolding proteins), Fe-S cluster scaffolding proteins (generation of Fe-S cofactors), and mitochondrial ferritin (iron sequestration) [1].

In a recent study published in *BMC Developmental Biology*, Metzendorf and Lind [2] establish *Drosophila *spermatogenesis as an emerging context for the study of mitochondrial iron metabolism through their analysis of the *Drosophila *gene *mitoferrin *(*dmfrn*). During spermatid development in flies, mitochondria undergo dramatic events of movement and shaping, including aggregation, fusion and elongation beside the growing flagellar axoneme. Disruptions of mitochondrial function or dynamics typically affect male fertility, a phenotype that is easily screened for and characterized at the subcellular level. The *dmfrn *gene product and other iron metabolism proteins show enriched expression in the testes of mammals and insects, suggesting special roles for mitochondrial iron metabolism in spermatogenesis. Analysis of these requirements in insect spermatogenesis could lead to new insights into human genetic disorders such as Friedreich's ataxia and sideroblastic anemia, both associated with defects in iron homeostasis within mitochondrial compartments.

Metzendorf and Lind characterized *D. melanogaster mitoferrin *(*dmfrn*) mutants and showed that the Dmfrn mitochondrial iron importer is required for spermatid mitochondrial morphogenesis and thus for development of mature motile sperm [2]. As in other invertebrates, the *Drosophila *genome includes a single homolog, *dmfrn*, of two human paralogs, *mitoferrin-1 *and *mitoferrin-2*, and two *Saccharomyces cerevisiae *paralogs, *mrs3 *and *mrs4*; all tests of cross-species complementation so far have indicated strong functional conservation. Studies in vertebrates and yeast demonstrate a role for mitoferrin family members in import of iron from the cytosol into mitochondria [3,4]. Metzendorf and Lind characterized three hypomorphic transposable element insertion alleles and two deficiency alleles affecting *dmfrn*. All alleles were associated with male sterility, and the deficiency alleles were associated with only a mild decrease in viability. The testis phenotype was enhanced and suppressed, respectively, by low and high iron levels in food.

**Figure 2:**
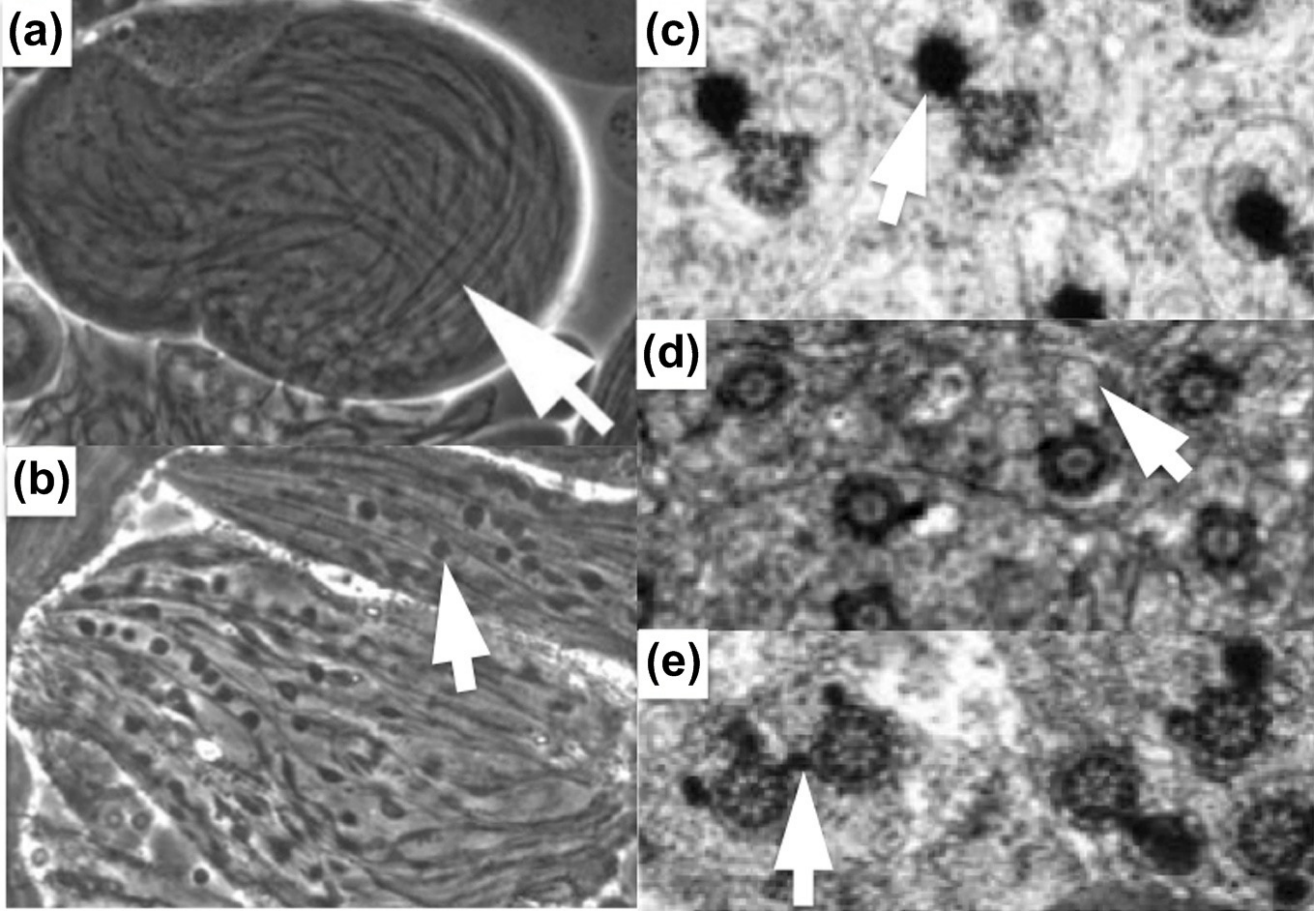
**The *dmfrn *gene is required for *Drosophila *male fertility and mitochondrial morphogenesis**. **(a, c) **Wild-type and **(b, d-e) ***dmfrn *elongating spermatids as visualized by phase-contrast microscopy (a, b) and transmission electron microscopy (c-e). Arrows in (a, b) indicate the elongating flagellum in (a) wild-type, and (b) abnormally bunched mitochondria in *dmfrn *elongating cells. The arrow in (c) indicates paracrystalline material of the wild-type major mitochondrial derivative adjacent to the pinwheel structure of the flagellar axoneme. (d) The arrow points to a *dmfrn *mitochondrial derivative devoid of any paracrystalline material. Some mutant *dmfrn *spermatids in (e) show aberrant paracrystalline material distribution in both mitochondrial derivatives. Figure adapted from [2].

The subcellular mutant phenotype in *dmfrn *testes was variable in severity, with some flies showing more robust testes and more frequent elongated cysts of spermatids than others. Failure of mitochondrial elongation accompanying axoneme elongation was common, as was failure of spermatid cyst individualization. In wild-type early round spermatids, the large, layered, spherical mitochondrial structure called the Nebenkern disentangles and elongates beside the nascent spermatid flagellar axoneme. In *dmfrn *mutants, the Nebenkern often remained aberrantly round while the axoneme microtubules grew (Figure 2). The failure of mitochondrial unfurling and elongation could be a simple result of low respiration and limited ATP to drive mitochondrial shaping. Alternatively, dmfrn could play a direct structural role in mitochondrial dynamics, analogous with the dual roles played by ATP synthase in both ATP synthesis and the shaping of the inner mitochondrial membrane. The phenotypic modulation of *dmfrn *by varying iron in the diet is more consistent with the former possibility.

At the ultrastructural level, as detected by transmission electron microscopy (TEM), *dmfrn *spermatid axonemes were associated with abnormally configured mitochondrial derivatives. Wild-type spermatid axonemes in TEM cross-section are accompanied by a major mitochondrial derivative containing electron-dense paracrystalline material; the minor mitochondrial derivative lacks this material and is mostly stripped away in the waste bag during individualization. Mutant *dmfrn *spermatids showed a striking decrease in paracrystalline material and aberrant distribution of what little was present, consistent with the speculation that iron may be a component of this yet-uncharacterized material (Figure 2). This paracrystalline material is proposed to provide a certain level of structural rigidity important for proper sperm movement before and during fertilization. The lack of paracrystalline material in *dmfrn *sperm cross-sections might alternatively simply reflect mitochondrial elongation failure.

Additional TEM data, combined with staining of actin and other structures, indicated that sperm individualization typically failed in *dmfrn *mutants. Individualization is the process at the end of spermatogenesis during which the 64 interconnected spermatids in a cyst become surrounded by their own plasma membranes. Failure of individualization is a typical secondary phenotype in mutant testes with an earlier structural defect.

The lack of dramatic phenotypes in tissues other than the testis led the authors to confirm by RT-PCR that *mitoferrin *showed enriched expression in testis tissue. A *dmfrn-lacZ *reporter construct indicated protein expression in post-meiotic spermatids. A tagged and functional dmfrn was associated with mitochondria in early round spermatids and later stages, with much of the protein sent to the waste bag during individualization. At the RNA level, the authors found similar testis enrichment of two additional iron-metabolism genes, *fh *and *Fer3HCH*. Expression patterns of these genes plus another, *Abcb10*, together suggest that *Drosophila *spermatogenesis could be a fruitful context for further analysis of iron metabolism.

The *Drosophila fh *gene is the ortholog of *S. cerevisiae YFH1 *and of human *FXN*, a gene associated with the neurodegenerative disorder Friedreich's ataxia. *FXN *encodes frataxin, a mitochondrial matrix protein whose role appears to be the delivery of iron to the scaffolding proteins on which Fe-S clusters are formed [4]. These Fe-S clusters later act as cofactors in many proteins important for cellular respiration. Frataxin may also play a role in the simple binding and detoxification of iron. Neurodegeneration in humans with Friedrich's ataxia stems from increased mitochondrial iron levels and neural oxidative damage; decreased levels of Fe-S-containing proteins presumably activate a pathway to boost mitoferrin-based iron import into mitochondria. *Drosophila *frataxin is expressed in multiple tissues besides the testis and, unlike mitoferrin, appears essential for normal viability. Studies of *Drosophila *frataxin are now possible through RNA interference [5]; perhaps future work will enable analysis in the testis.

In mammals, the ABC-family transporter protein Abcb10 forms a complex with mitoferrin-1 and increases iron import into mitochondria, as recently characterized in erythroid tissue [6]. A close homolog in *Drosophila *is evident through sequence homology searches of the *Drosophila *genome [7]; the FlyAtlas database reports that this gene shows enriched expression in both testes and ovaries in *Drosophila *[8]. Analysis of the fly version of *Abcb10 *may enable exploration of genetic interactions with *dmfrn *and the physical interactions of their gene products. Ferritin is an iron-sequestration protein that has been extensively studied in its cytosolic and secreted forms. Recent years have seen the discovery of a new ferritin in mammals and insects that is targeted to mitochondria. *Drosophila *mitochondrial ferritin, encoded by *Fer3HCH*, is highly expressed in the testis, and the gene product localizes to mitochondria when transfected into human cells [9]. This gene is ripe for analysis with regard to its role in spermatogenesis and its interactions with *mitoferrin*, *fh*, and *Abcb10*. Mammalian mitochondrial ferritin is similarly expressed highly in testes [10]; broader studies of human male fertility may in the future lead to a focus on iron metabolism in developing sperm.

Metzendorf and Lind's identification of a role for the mitochondrial iron importer dmfrn in *Drosophila *sperm development, and their confirmation of testis expression of other proteins involved in iron homeostasis, set the stage for future exploration of iron metabolism as an important factor in mitochondrial morphogenesis and fertility in both vertebrates and invertebrates.
